# Electronic and optical properties of bulk and surface of CsPbBr_3_ inorganic halide perovskite a first principles DFT 1/2 approach

**DOI:** 10.1038/s41598-021-99551-y

**Published:** 2021-10-18

**Authors:** Mohammed Ezzeldien, Samah Al-Qaisi, Z. A. Alrowaili, Meshal Alzaid, E. Maskar, A. Es-Smairi, Tuan V. Vu, D. P. Rai

**Affiliations:** 1 Physics Department, College of Science, Jouf University, P.O. Box 2014, Sakaka, Al-Jouf Saudi Arabia; 2 Palestinian Ministry of Education and Higher Education, Nablus, Palestine; 3 Nanomaterial and Nanotechnology Unit, E. N. S. Rabat, Energy Research Center, Faculty of Sciences, Mohammed V University in Rabat, B.P. 1014, Rabat, Morocco; 4 Laboratory of Physics of Condensed Matters and Renewables Energies, Hassan II University, Faculty of Sciences and Technologies, B.P 146, 20650 Mohammedia, Morocco; 5 Division of Computational Physics, Institute for Computational Science, Ton Duc Thang University, Ho Chi Minh City, Vietnam; 6 Physical Sciences Research Center (PSRC), Department of Physics, Pachhunga University College, Mizoram University, Aizawl, India

**Keywords:** Condensed-matter physics, Materials for devices, Materials for optics, Theory and computation

## Abstract

This work aims to test the effectiveness of newly developed DFT-1/2 functional in calculating the electronic and optical properties of inorganic lead halide perovskites CsPbBr_3_. Herein, from DFT-1/2 we have obtained the direct band gap of 2.36 eV and 3.82 eV for orthorhombic bulk and 001-surface, respectively. The calculated energy band gap is in qualitative agreement with the experimental findings. The bandgap of ultra-thin film of CsPbBr_3_ is found to be 3.82 eV, which is more than the expected range 1.23-3.10 eV. However, we have found that the bandgap can be reduced by increasing the surface thickness. Thus, the system under investigation looks promising for optoelectronic and photocatalysis applications, due to the bandgap matching and high optical absorption in UV–Vis (Ultra violet and visible spectrum) range of electro-magnetic(em) radiation.

## Introduction

In recent years, the organic-inorganic hybrid metal-halide perovskite compounds based photovoltaics (converts sunlight into usable electricity) have garnered considerable scientific interest owing to their low cost fabrication via simple solution-based method and high power conversion efficiency (PCE)^1^. The less expenditure and simple preparation technique from the standard lab equipments enabled the mass-scale production of Methylammonium lead Iodide (CH_3_NH_3_PbI_3_), and finally lead to extensive scientific research. The organic-inorganic halide perovskites are denoted by a general single perovskite formula ABX_3_ generally A is an organic cation(CH_3_NH$_{3}^+$), B is divalent metal (Pb$^{2+}$, Sn$^{2+}$, Ge$^{2+}$, Mg$^{2+}$, and Ca$^{2+}$), and X is a halide ion (Cl$^-$, Br$^-$ and I$^-$)^2^. Recently, it has been reported a huge leap in the progress of PCE up to 23.3% from 3.8% in methylammonium lead iodide organic-inorganic hybrid perovskite compound (CH_3_NH_3_PbI_3_)^3,4^. This unprecedentedly high value of PCE has been attributed to high bipolar charge carrier mobility ($\sim$ 0.6 cm$^2$V$^-1$s$^1$)^5^, high absorption coefficient in visible light range, tunable bandgap, strong fluorescence quantum yield, balanced charge mobility (electron-hole), low rate of electron-hole pair recombination, high carrier lifetimes, and high diffusion lengths^6–10^. Also, the presence of an extraordinary optoelectronic properties in organic-inorganic hybrid lead-halide perovskite compounds have a wide application in photonics like light-emitting diodes, and lasers^8,11–14^. It was reported the tunability of bandgap (E$_g$) from 1.6 to 2.3 eV in MAPb (I$_{3-x}$Br$_x$) [CH$_3$NH$_{3}^+$ = Methylammonium (MA)] and a PCE of 12.3% under standard AM-1.5^15,16^ (AM = air mass). But with the Sn doping the band gap reduces to $\sim$1.2 eV^17^. Despite, its cost-effectiveness and high photovoltaic absorbers efficiency, organic-inorganic hybrid halide perovskites have limited practical/commercial uses due to their low stability as a consequence of surface oxidation when exposed to the environment (moisture/humidity)^3^. It has been reported that chemical instability is an intrinsic property of CH$_3$NH$_3$PbI$_3$^18^. The instability in CH$_3$NH$_3$PbI$_3$ can be eliminated by doping Br at I-site^16^. Liu et al., investigated the cation doping of aziridinium (Az$^+$) on CsPbI$_3$ from first-principles DFT (Density Functional Theory) and reported the improved stability with the widening of the bandgap from 1.76 to 2.27 eV^19^.

Additionally, solid-state inorganic metal-halide perovskite materials are promising with high stability at ambient condition^20–23^ as compared to organic-inorganic hybrid halide perovskites. The solid-state inorganic single perovskite materials have the chemical formula of AMX$_3$, where A is a cation (Li, Na, K, Rb, Cs, etc.), M is another cation (typically Pb/Sn) and X is a halide (F, Cl, Br, I). Almost, all-inorganic halide perovskites such as CsPbBr$_3$, CsPbI$_3$, and their alloys [CsPbBr$_x$I$_{3-x}$] are shown to have greater stability as compared to CH$_3$NH$_3$PbI$_3$, while their optoelectronic properties are in line with organic-inorganic hybrids (CH$_3$NH$_3$PbI$_3$)^24,25^. The long-term stability of inorganic solid-state perovskite materials are the primary impediment to their widespread implementation. A single inorganic perovskite compounds APbBr$_3$ (where A is Li, Na, K, Rb, and Cs) display semiconducting characteristics with energy bandgap in the range of 1.708–1.769 eV^6^. Recently, McGrath et al., synthesised highly stable nanocrystals of the inorganic halide perovskites CsPbBr$_3$ employing oleylamine/alkylphosphonic acid displaying outstanding photo-physical and chemical properties^26^. At the experimental level, perovskite/silicon solar cells are in tandem with enhanced efficiencies when compared with other perovskite-based multi-junctions. Beal et al., reported an improved chemical-stability and efficiency up to 10.77% for tandem solar cells based on solid state inorganic CsPbI$_3$ perovskite^27^. Ouedraogo et al.^28^, have suggested the different strategies and fabrication method to improve the stability of black phase of CsPbI$_3$ such as Solvent-additives engineering, Alloying/element doping engineering, 2D nanocrystal engineering. On the other hand, calorimetric investigations on the formation enthalpies of CsPbX$_3$ perovskites have shown the diminishing order of thermodynamic stability for CsPbCl$_3$, CsPbBr$_3$, and CsPbI$_3$^29^. From various studies it has been reported that CsPbBr$_3$ undergoes structural phase transitions from orthorhombic to tetragonal and subsequently to cubic at higher temperatures $\left( \xrightarrow [< 80 \, ^{\circ } \text {C}]{\text {orthorhombic}}\xrightarrow [80\, ^{\circ } \text {C}-130 \, ^{\circ } \text {C}]{\text {tetragonal}}\xrightarrow [130 \, ^{\circ } \text {C}<]{\text {cubic}}\right)$^30–36^.

Apart from their superior bulk properties, it is highly crucial to preserve such intriguing properties at their surfaces and interfaces for device applications. Surfaces and interfaces play a key role in device fabrications and deciding the device’s performance. Thus it needs the critical understanding and thorough knowledge of the surface characteristics of inorganic perovskites. A substantial influence of surface phenomena (surface energy, atomic structures, and electronic structures, etc.) on material stability and device performance has been seen in nanostructures due to their high surface-to-volume ratio. The influence of surface energy on the stability of CsPbX_3_ (X = Cl, Br, I) nanocrystals^37^ and stability of meta-stable nanocrystal via strain effect have been reported in several studies^38–40^. The surface-guided growth techniques have been used to synthesise the CsPbBr$_3$ nanowires^41^. The experimental study has identified that 2D CsPbBr$_3$ with CsBr-terminated (100) surface is the most stable one^41,42^. Theoretically, the first principles study using VASP (Vienna Ab initio Simulation Package) has reconfirmed the ground state stability of non-polar CsBr-terminated (100) surface as compared to (110), and (111) polar-surfaces^43^.

A first principles DFT^44^ is used to comprehend the experimental findings and gain insight into atomistic-scale interactions in deriving the varied physical properties of a material. Ghaithan et al., performed a fist principles DFT calculation on CsPbBr$_{3-x}$Cl$_x$ Perovskite using PBE-GGA and mBJ-GGA functional^45^. They have reported an enhanced band gap from 2.23 to 2.90 eV with increasing Cl concentration within the modified Becke-Johnson generalized gradient approximation (mBJ-GGA) potential^45,46^ Here, in this study we concentrate on the bulk orthorhombic phase of CsPbBr$_3$ which exist below 80 °C may be favourable for ground state DFT based first-principles calculation. We have also performed the ground state calculation on the surface stability of orthorhombic CsPbBr$_3$.

## Computational details

For computation of both the bulk and surface of CsPbBr$_3$ we have used Kohn-Sham DFT (KS-DFT) based *Atomistic Simulation Software QuantumATK (VNL-ATK)* which incorporate Linear Combination of Atomic Orbital (LCAO) basis function^47,48^. All electrons are treated by a newly modified potential ($V_S$) that has been developed by correcting the self-interaction error within the exchange-correlation functional *via* semi-empirical approach often denoted as DFT-1/2^49,50^. In this approach the general effective potential $V_{eff}$ in KS-DFT equation has been substituted to obtain a modified potential $V_{mod}$=$V_{eff}- V_S$. The corrected term $V_S$ is analogous to the half-occupation state of Slater scheme, mimics the electrostatic potential of the atoms in the crystal. Since it is impossible to sum all the divergent potentials for infinite periodic lattices, the extended part is truncated using the step function.

1 $$\begin{aligned} \Theta (r)&= \left\{ \begin{array}{ll} A\left[ 1-\left( \frac{r}{r_{\mathrm{cut}}} \right) ^{\!\!n} \right] ^3 &{} r \le r_{\mathrm{cut}} \\ 0 &{} r>r_{\mathrm{cut}} \end{array} \right. \end{aligned}$$

2 $$\begin{aligned} V_S(r)\rightarrow V'_S(r)&= \Theta (r)V_S(r) \end{aligned}$$

**Figure 1:**
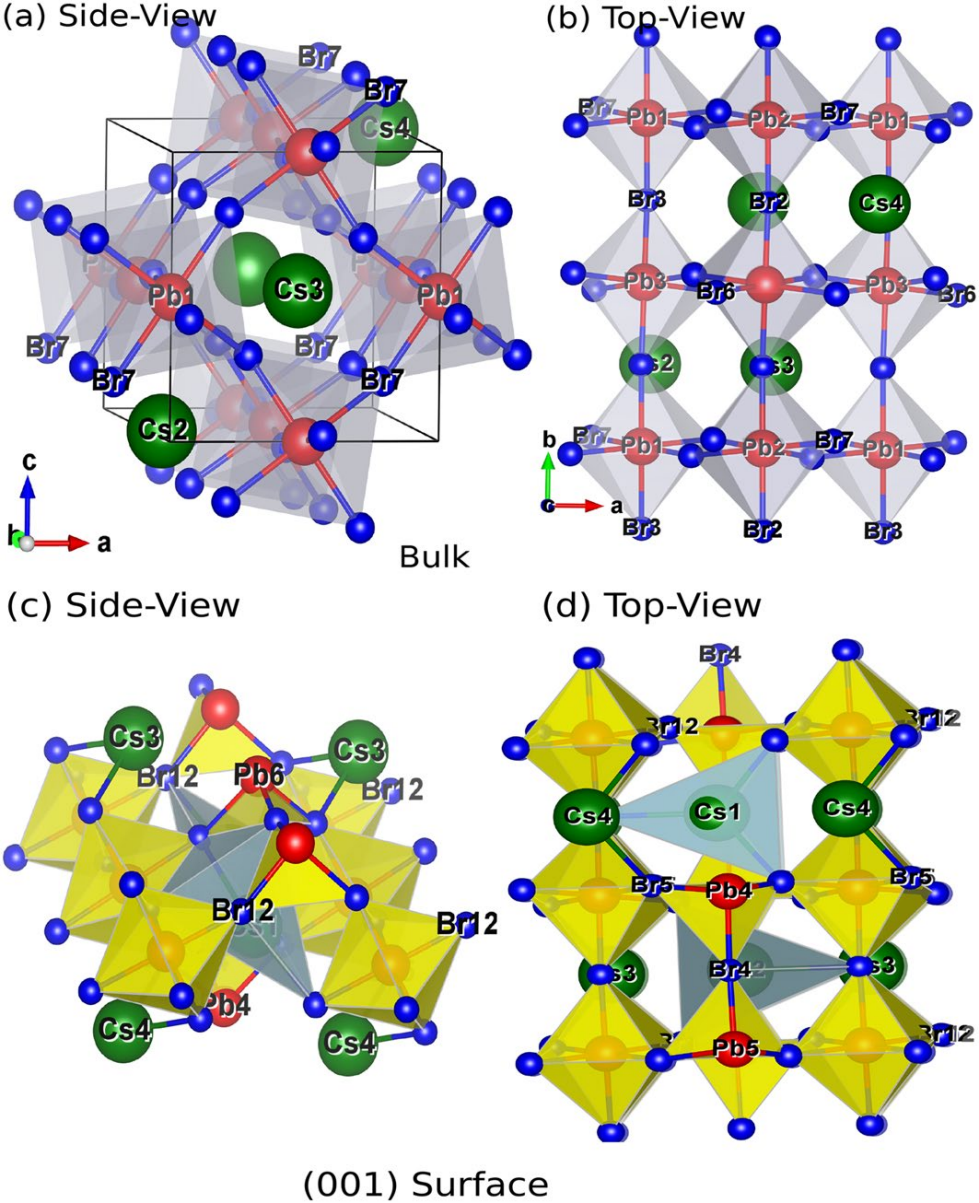
(**a**, **b**) Top and Side View of orthorhombic CsPbBr_3_ and (**c**, **d**) Top and Side View of 001-surface of CsPbBr_3_ along with polyhedra cage (Cs-green, Pb-red, Br-blue).

Here, cut off radius $r_{cut}$ estimated variationally, $n=8$ and *A* is the amplitude of the correction. The corrected term $\Theta (r)V_S(r)$ is confined within a sphere of radius $r_{cut}$, enable its application in band-structure calculation. This means DFT-1/2 is plausible for improving the degenerated semiconductor bandgap up to 10 eV^49,50^. Tao et al. has reported the performance of DFT-1/2 in opening the bandgap in metal halide perovskites, comparable to that of GW^51^. Moreover, we are familiar with DFT-1/2 approach and reported increased band gap in our previous studies of 1D (6,1) single walled Carbon nanotube^52^ and 2D hexagonal ZnSe^53^. At low temperature CsPbBr$_3$ crystallizes in orthorhombic phase having space group *Pnma*. An ultra-thin 001-surface structure has been cleaved using $2\times 2\times 1$ supercell with the repetition of unit cell along x and y direction. A vacuum of 15 (Å) is imposed along the z-axis to interrupt the lattice periodicity which eventually avoid nonphysical interaction of the wave functions. We have considered the bulk and surface of orthorhombic CsPbBr_3_ and structural optimization was performed from PBE-GGA^54^, rather than DFT-1/2 due to its limitation in calculating the total energy. The orthorhombic crystal of CsPbBr_3_ and 001 surface are presented in Fig. 1a–d.

## Result and discussion

The energy-volume curve is obtained by fitting the data obtained from first principles self-consistent force (SCF) calculation in the third order Murnaghan equation of state^55^ as

3 $$\begin{aligned} E(V)=E_o+\frac{9V_oB_o}{16}\Big [ \left[ (V_o/V)^{2/3}-1 \right] ^3B'_o+ \left[ (V_o/V)^{2/3}-1 \right] ^2 \left[ 6-4(V_o/V)^{2/3} \right] \Big ] \end{aligned}$$

**Figure 2:**
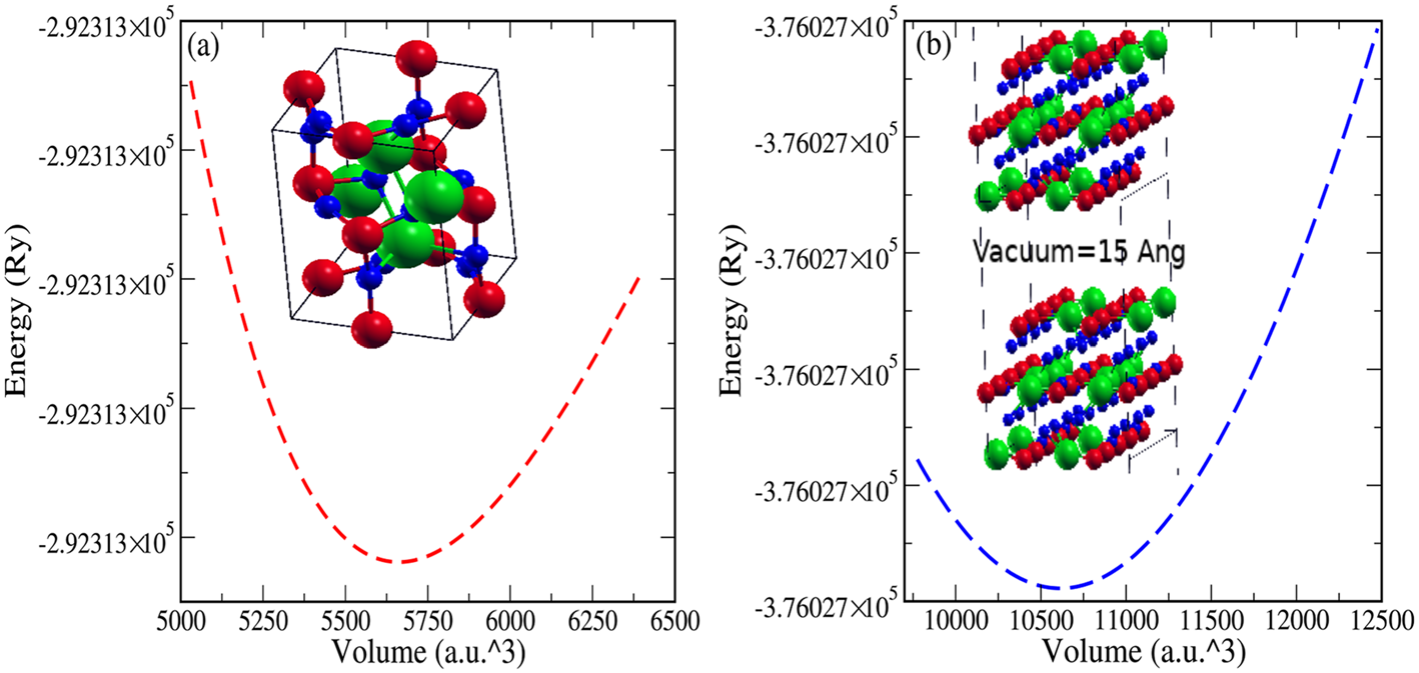
Variation of Ground state energy and Volume (V) for (**a**) Bulk and (**b**) Surface of CsPbBr$_3$ (Cs-green, Pb-red, Br-blue).

The volume optimization curve for both the bulk and the surface is shown in Fig. 2a and b. The atomic positions, previous experimental/theoretical lattice constants^45,56^ and optimized lattice constants are presented in Table 1. Our calculated lattice constant varies by a very small amount, $\frac{a_{expt}-a_{th}}{a_{expt}}\times 100=\sim 0.3\%$. The lattice constants of the surface after symmetry operation has become $a=8.135$ (Å) and $b=11.597$ (Å) taking the space group P21/m. While after volume optimization, the optimized lattice constants for 001-surface are $a=8.369$ (Å) and $b=11.931$ (Å). The bulk modulus (B) is the natural output of volume-optimization. The calculated bulk modulus $B=20.95$ GPa in good agreement with the previous results 26.841 (LDA)^45^, 19.203 (GGA)^45^, 22.759 (PBE-sol)^45^, 21.56^57^ and 18 GPa^56^. We also report the bulk modulus of surface CsPbBr_3_ $\sim$ 9.618 GPa which is almost half of its bulk counterpart.

**Table 1:** Atomic positions, experimental lattice constants and theoretical (calculated in this work) lattice constants of orthorhombic bulk CsPbBr_3_.

Atom	Expt. position			Expt. lat const. (Å)			Th. lat const. (Å)		
	*x*	*y*	*z*	$a_{expt}$	$b_{expt}$	$c_{expt}$	$a_{th}$	$b_{th}$	$c_{th}$
Cs	0.008	0.032	0.750	8.370	12.011	8.425^a^	8.343	11.973	8.398
	0.492	0.532	0.750	8.561	12.187	8.514^b^			
	0.508	0.468	0.250						
	0.992	0.968	0.250						
Pb	0.000	0.500	0.000						
	0.000	0.500	0.500						
	0.500	0.000	0.500						
	0.500	0.000	0.000						
Br	0.048	0.506	0.250						
	0.205	0.795	0.974						
	0.205	0.795	0.526						
	0.295	0.295	0.974						
	0.295	0.295	0.526						
	0.452	0.006	0.250						
	0.548	0.994	0.750						
	0.705	0.705	0.026						
	0.705	0.705	0.474						
	0.795	0.205	0.026						
	0.795	0.205	0.474						
	0.952	0.494	0.750						

^a^^56^, ^b^^46^.

### Electronic properties

**Figure 3:**
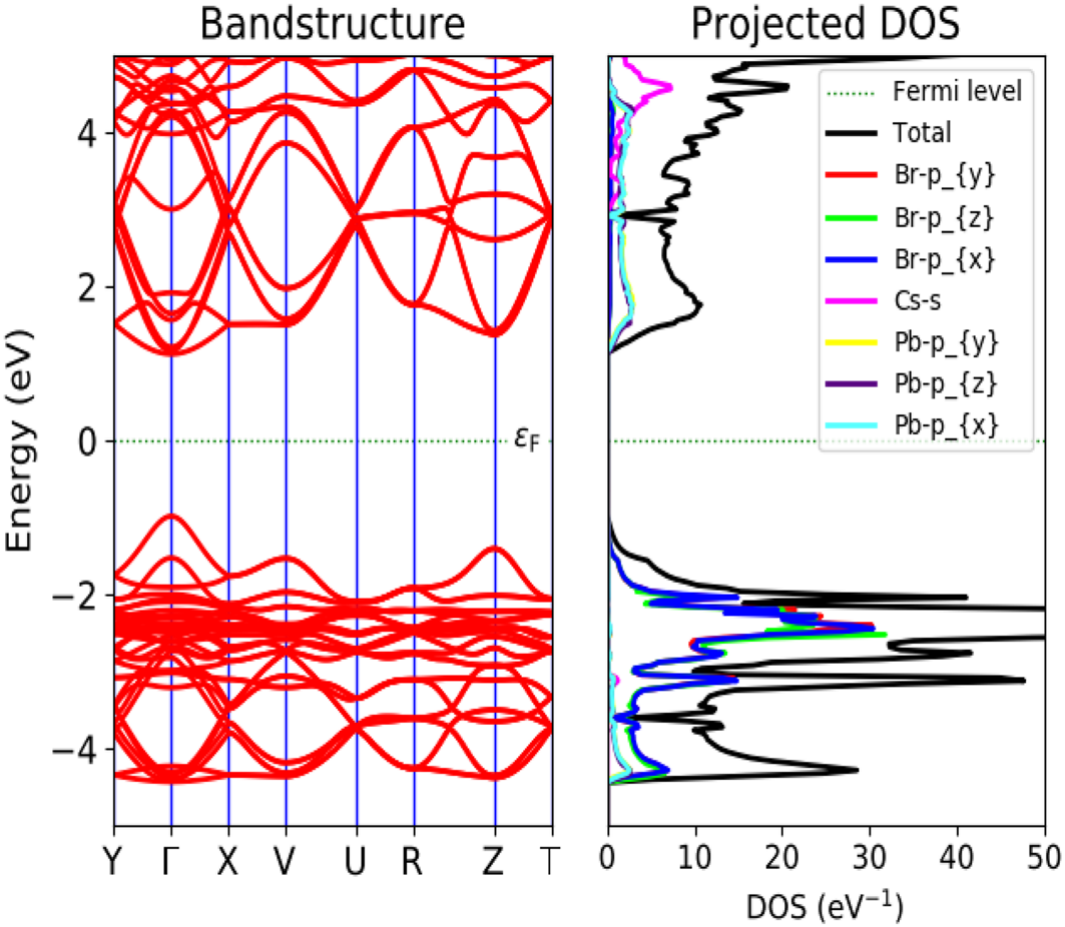
Band structure and density of states (DOS) of bulk orthorhombic CsPbBr$_3$ calculated from DFT-1/2.

**Figure 4:**
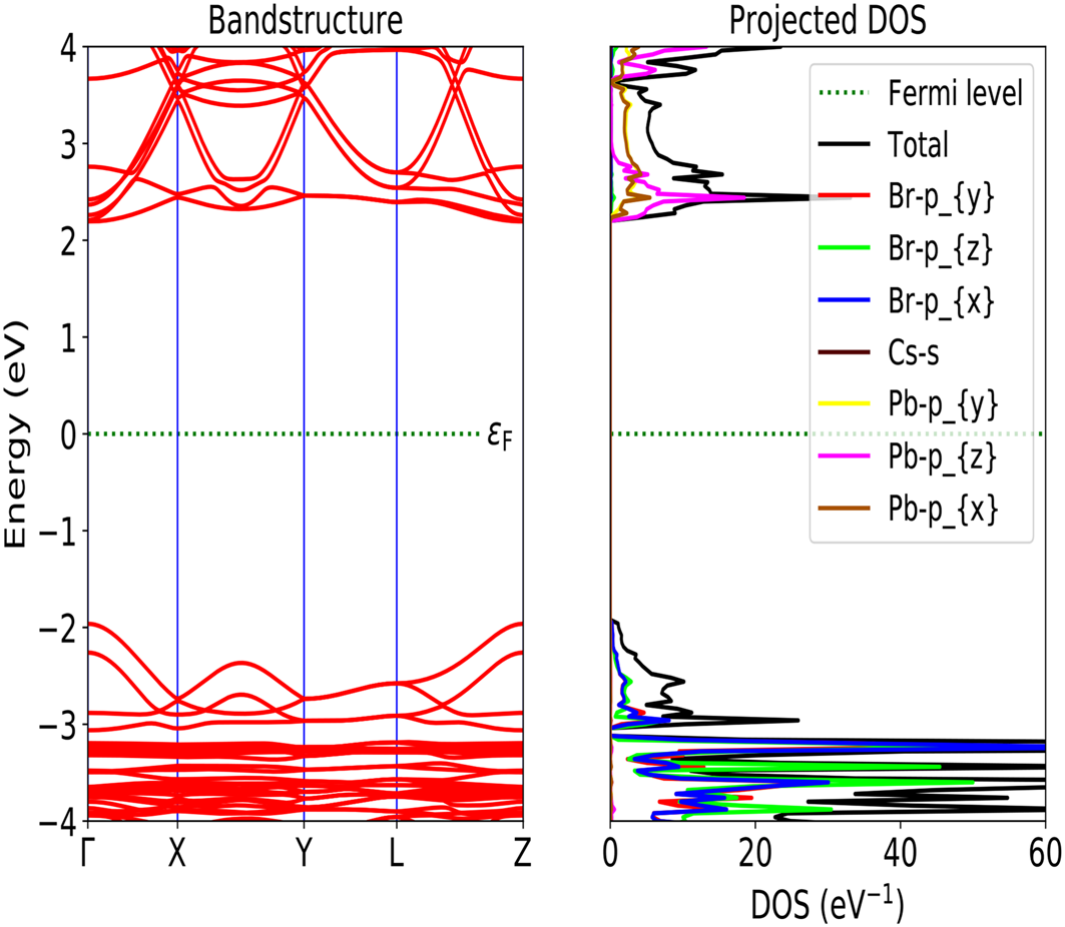
Band structure and density of states (DOS) of 001-surface of CrPbBr$_3$ calculated from DFT-1/2.

**Figure 5:**
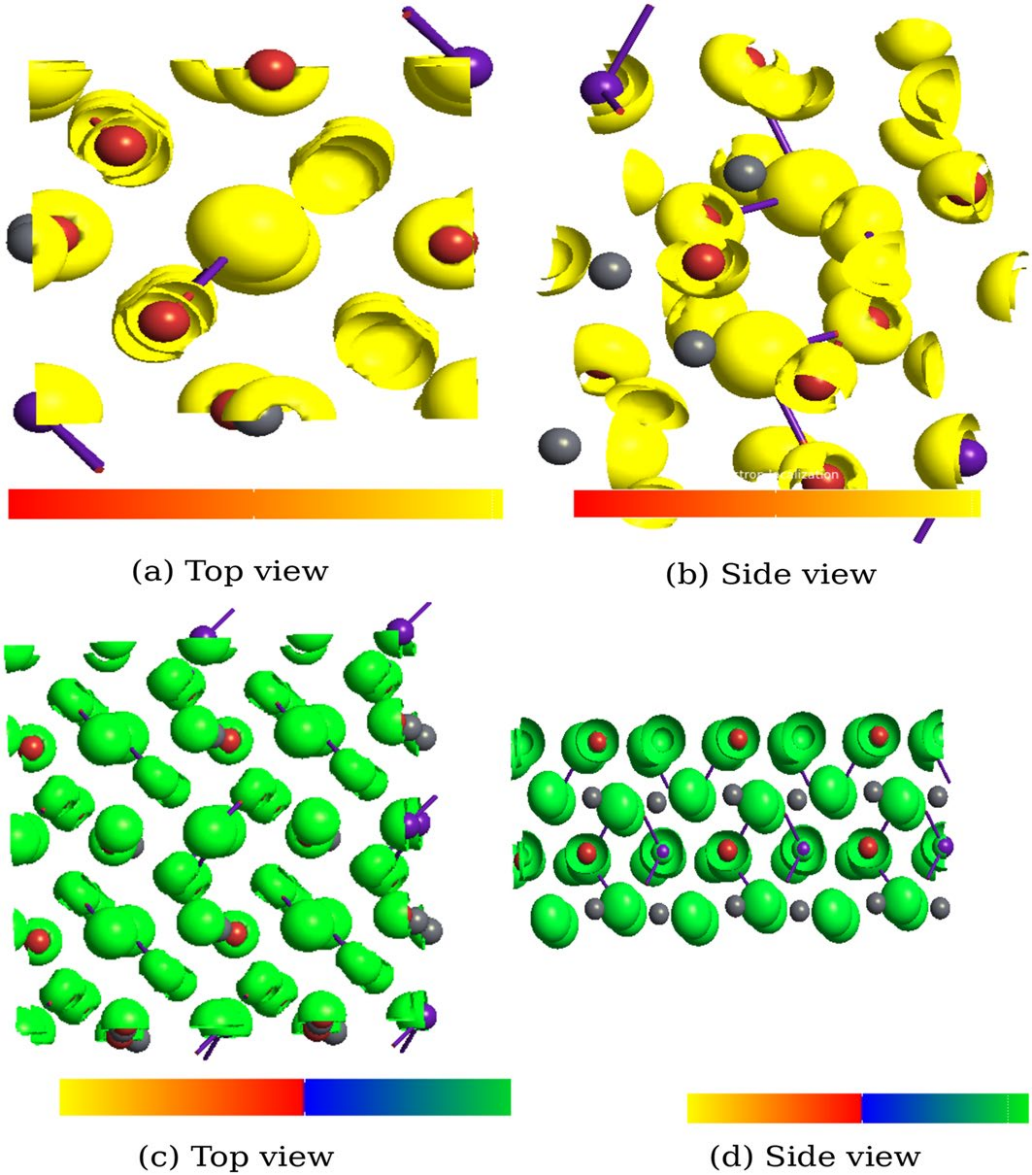
(**a**, **b**) Top-side view of 3D electron density map of bulk orthorhombic CrPbBr$_3$ and (**c**, **d**) Top-side view of 3D electron density map of 001-surface of CsPbBr$_3$.

The optimized structure has been used for the calculation of electronic and optical properties of both the bulk and surface structure from DFT-1/2 approach. The importance of DFT-1/2 in predicting accurate electronic bandgap ($\le$10 eV) and convergence feasibility has already been discussed. In our study both bulk and surface structure exhibit a semiconducting behaviour with a bandgap of $\sim$ 2.36 eV and $\sim$ 3.82 eV, respectively (see Figs. 3, 4). The presence of Fermi level (E$_F$) at the middle of the bandgap indicate its intrinsic behaviour with a direct bandgap along $\Gamma -\Gamma$ symmetry. Our result of electronic band-gaps calculated from semi-local DFT-1/2 are in consistent with the previous results obtained from higher order DFT functional like GW, HSE, mBJ etc., and experiment [for numerical comparison see Table 2]. Meanwhile, DFT-1/2 open up the the underestimated bandgap from GGA and local density approximation (LDA) by $\sim$ 20%. Our result of bandgap (2.36 eV) for orthorhombic CsPbBr$_3$ from DFT-1/2 approximation is in consistent with the experimental value of 2.23 eV^58^, 2.446 eV^59^ and 2.36 eV^60^. It also agrees well with the result (2.28 eV) of higher order DFT like HSE03^61^. Hussain et al., reported the reduced band gap of 1.27 eV, 1.16 eV and 1.08 eV for the cubic, tetragonal and orthorhombic phases, respectively, using spin-orbit coupling in WIEN2K code^62^. While Yang has reported 1.11 eV for the cubic phase incorporating spin-orbit coupling in VASP package^63^ From Fig. 3, we have observed that the top of the valence band is mainly composed of Cs-*s* and Pb-*p*. It clearly shows the *s*-state of Cs atom overlap with the $p_x$ state of Pb atom indicating a strong $s-p$ hybridization, while the non-bonding states are formed at the conduction region far above the Fermi level. The Br-*p* and Pb-*p* states form a covalent bond. While the Cs-*s* and Br-*p* gives a weak ionic bonding. The majority contribution at the bottom of the conduction band merely comes from the Br-*p* states and empty Pb-*p* states. The two-fold degenerated bands along the $\Gamma$ symmetry, infer the presence of heavy and light effective masses of electrons, already discussed elsewhere^45^. Figure 5a and b, shows the top and side view of 3D electron density plot of bulk orthorhombic CsPbBr$_3$. Figure 5c and d, displays the top and side view of 3D electron density plot of 001-surface of CsPbBr$_3$. The localization of density is given by yellow sphere around the Pb and Cs atoms.

**Table 2:** Result of electronic bandgap (E$_g$) in eV of bulk and surface of CsPbBr$_3$ from DFT-1/2 and comparison with the results of other functional.

Functional	Cubic	Tetragonal	Orthorhombic	Surface
DFT-1/2	–	–	2.36	3.82
PBE-GGA	1.40^a^,1.76^b^,1.6^f^	1.49^b^	1.78^b^, 2.00^c^, 2.29^g^	1.78^a^
			2.154^d^	
EV-GGA	2.10^b^, 1.764^e^	1.92^b^, 2.3^d^	2.16^b^, 2.11^g^	–
PBEsol-GGA	1.65^b^, 2.633^h^	1.40^b^, 1.9^i^	1.68^b^, 2.60^g^	–
mBJ-GGA	2.66^b^, 2.36^d^	2.35^b^	2.58^b^, 3.4^g^, 2.23^d^	–
mBJ-GGA-SO	1.81^b^	1.69^b^	1.82^b^, 2.73^d^	–
HSE6-SO	2.74^j^	–	–	–
Expt.	2.30^k^		2.36^n^	2.84^l^/2.3^p^
	2.36^l^		2.24^n^	2.93^q^
	2.32^m^		2.32^o^	

^a^^43^, ^b^^45,46^, ^c^^56^, ^d^^33^, ^e^^64^, ^f^^65^, ^g^^66^, ^h^^67^, ^i^^68^, ^j^^57^, ^k^^69^, ^l^^70^, ^m^^71^, ^n^^60^, ^o^^72^, ^p^^73^, ^q^^59^.

**Figure 6:**
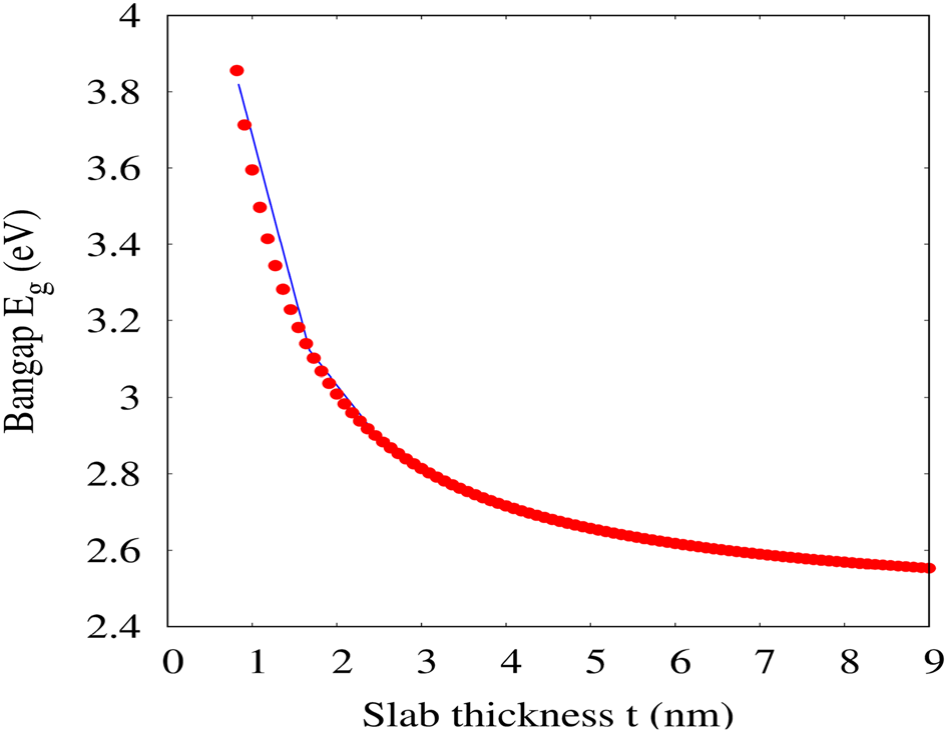
The variation of energy bandgap (E$_g$) as a function of slab thickness (t) of 001-surface of CrPbBr$_3$ using DFT-1/2 (blue line represent the results of energy bandgap and red dot denotes the data fit using Eq. (4) from gnuplot).

On the other hand the exfoliation of ultra-thin 001-surface of CsPbBr$_3$ shows the large opening of band gap by $\sim$42% as compared to the bulk. Despite, widening of the band gap of the 001-surface, the electronic band morphology has been preserved with its direct band gap along $\Gamma -\Gamma$ symmetry. Similar to the bulk, we have noticed a coupling between Pb-Br, and Cs-Br ions, suggesting covalent and ionic bond, respectively, in surface as well (see Fig. 4). Brescia et al.^59^, formulated an expression for theoretical prediction of onset band gap by fitting a power function with a set of experimentally determined bandgap as a function of film thickness $'t'$ in relation to effective mass $m^*$.

4 $$\begin{aligned} E_g^t=E_g^{bulk}+\frac{\hbar ^2\pi ^2}{2m^* t} \end{aligned}$$

where $E_g^{bulk}$ and $E_g^t$ are the energy band gap of bulk and film of thickness (*t*) respectively. It is obvious from the above expression that the decrease in film thickness will increase the energy band gap of the thin film. In Fig. 6, we have presented the variation of energy bandgap as a funtion of slab thickness of CsPbBr$_3$. The curve is fitted based on the Eq. (4) using gnuplot. The similar trend of increase in band gap from 2.47 eV to 2.84 eV on decreasing the slab from 15$\rightarrow$1 layer has also been reported from PWscf PBE-SOC (spin-orbit coupling) calculation^70^.

The increased in the direct band gap of the surface state may be infer to quantum confinement effect^74^. The halide based nanoparticles perovskite exhibit quantum confinement effect for a nanometer-size (< 50 nm) and strong Mie resonances above 10^2^ nm^75^. We have arbitrarily determine the electron effective mass of 001-surface using Eq. (4), taking the single layer thickness $t=8.398$ Å, $E_g^t=3.82$ eV and $E_g^{bulk}=2.36$ eV. The estimated electron effective mass of 001-surface is found to be $\sim$0.20$m_e$, in good agreement with 0.24$m_e$ (single slab) and 0.17$m_e$ (bulk)^70^.

### Optical properties

For better explanation of the optical response the electron transitions are considered from the first three valence bands below E$_F$ to first three conduction bands above the E$_F$. The probability transition along the various symmetry points in the first Brillouin zone and their corresponding maximum energies give the information about the optical properties. The optical response with respect to the incident photon energy is presented in the form of a complex dielectric function given by;

5 $$\begin{aligned} \varepsilon = \varepsilon _{1} + \iota \varepsilon _{2}, \end{aligned}$$

Here $\varepsilon _{1}$ and $\varepsilon _{2}$ are real and imaginary parts of the dielectric function respectively. The imaginary part can be calculated using Eq. (2),

6 $$\begin{aligned} {\varepsilon _{2} (\omega )}= & {} \dfrac{\hbar ^{2}{e^{2}}}{{\pi }{m^{2}}{\omega ^{2}}}\Sigma _{nn^{'}}\int _k {d^{3}k}|<\overrightarrow{k}n|\overrightarrow{p}|\overrightarrow{k}n^{'}>|^{2} [1-f(\overrightarrow{k}n)]\nonumber \\&\quad \delta (E_{\overrightarrow{k}n}-E_{\overrightarrow{k}n^{'}}-\hbar \omega ), \end{aligned}$$

where $\overrightarrow{p}$ - momentum operator, $|{\overrightarrow{k}n}>$ - eigen-function of eigenvalue, f($\overrightarrow{k}n$)-Fermi Distribution function. The Kramers–Kronig transformation helps in finding the real part of the dielectric function from its corresponding imaginary part as :

7 $$\begin{aligned} {\varepsilon _{1}(\omega )} = 1 + \dfrac{2}{\pi } \int _{0}^{\infty } \dfrac{\varepsilon _{2}(\omega '){\omega '}d\omega '}{\omega ^{'2}-\omega ^{2}}, \end{aligned}$$

The absorption coefficients $\alpha (\omega )$, which is related to the dielectric function is given as follows;

8 $$\begin{aligned} \alpha (\omega ) = \dfrac{2\omega (|\varepsilon (\omega )|-Re \varepsilon (\omega ))^{1/2}}{c} \end{aligned}$$

The real part of refractive index (*n*) is given by

9 $$\begin{aligned} n=\sqrt{\frac{1}{2}\Big ( (\varepsilon _1^2+\varepsilon _2^2)^{1/2}+\varepsilon _1 \Big )} \end{aligned}$$

**Figure 7:**
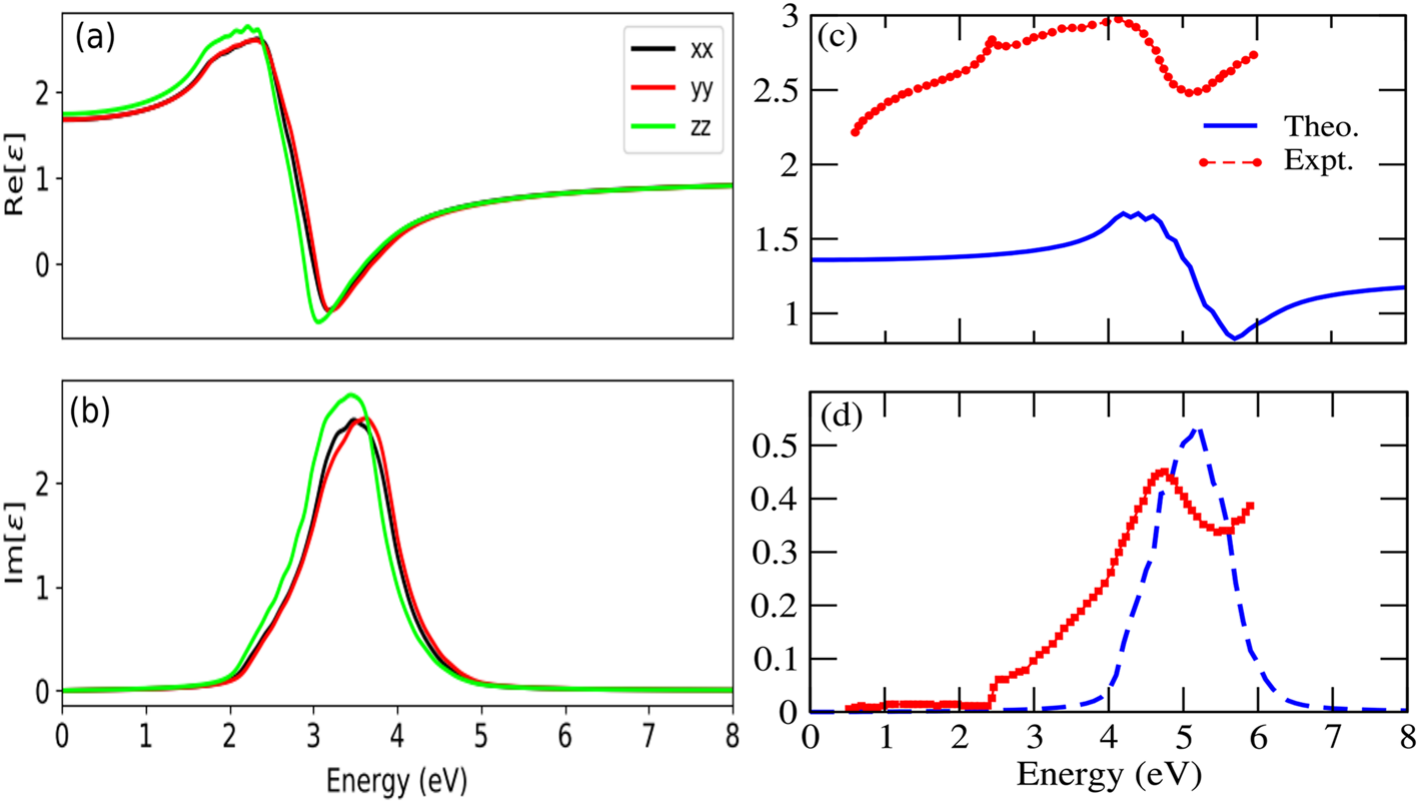
(**a**) Real part of dielectric function ($\varepsilon _1$) of bulk (**b**) Imaginary part of dielectric function ($\varepsilon _2$) of bulk, (**c**) Real part of dielectric function ($\varepsilon _1$) of 001-surface (**d**) Imaginary part of dielectric function ($\varepsilon _2$) of 001-surface, [*our result is compared with the experimental spectra of $\varepsilon _1$ and $\varepsilon _2$(denoted by red dot) Expt.^76^].

**Figure 8:**
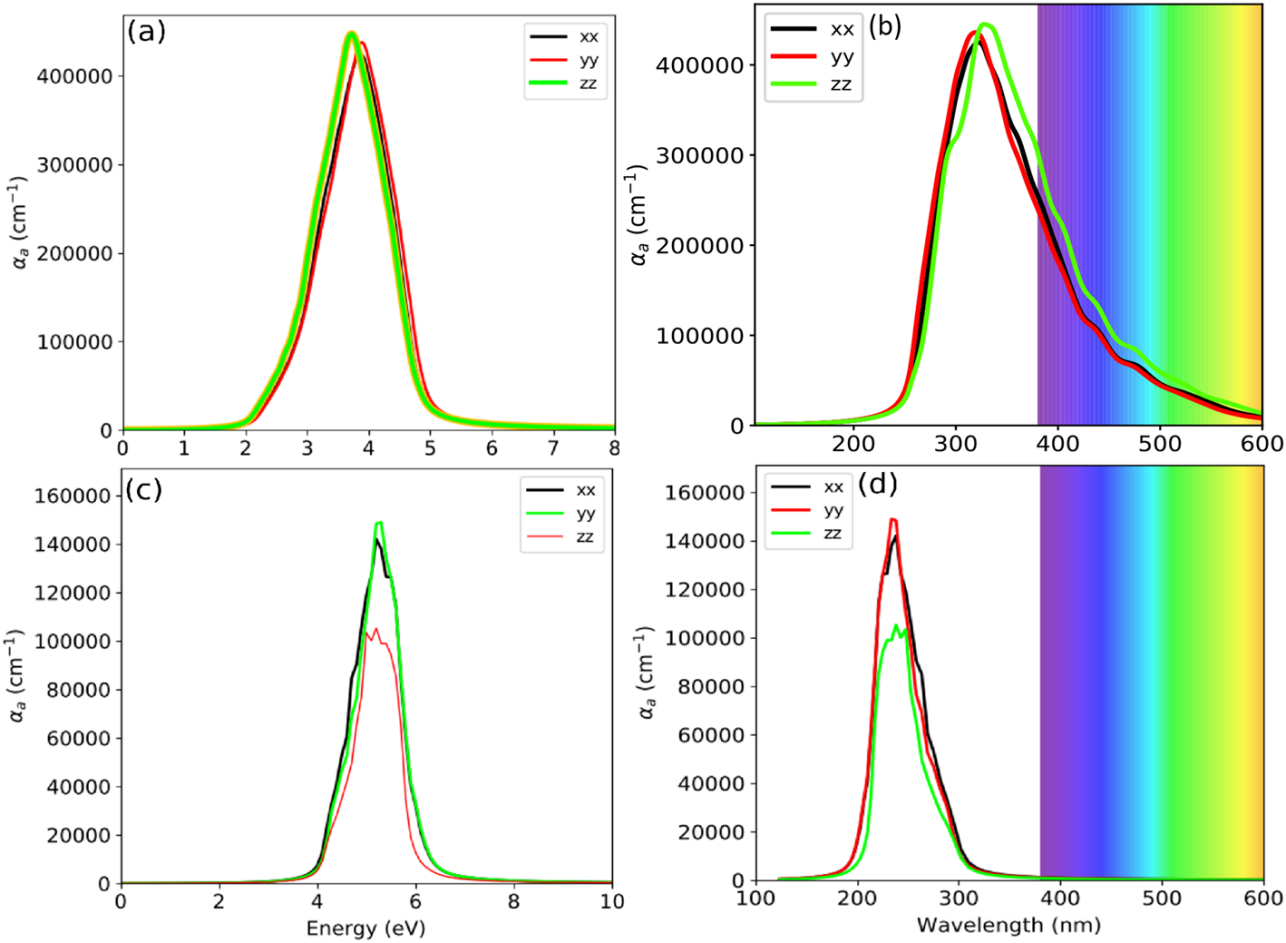
Absorption coefficient ($\alpha _a$) of bulk with respect to (**a**) Photon energy (eV) (**b**) Wavelength (nm), and Absorption coefficient ($\alpha _a$) of 001-surface with respect to (**c**) Photon energy (eV) (**d**) Wavelength (nm).

**Figure 9:**
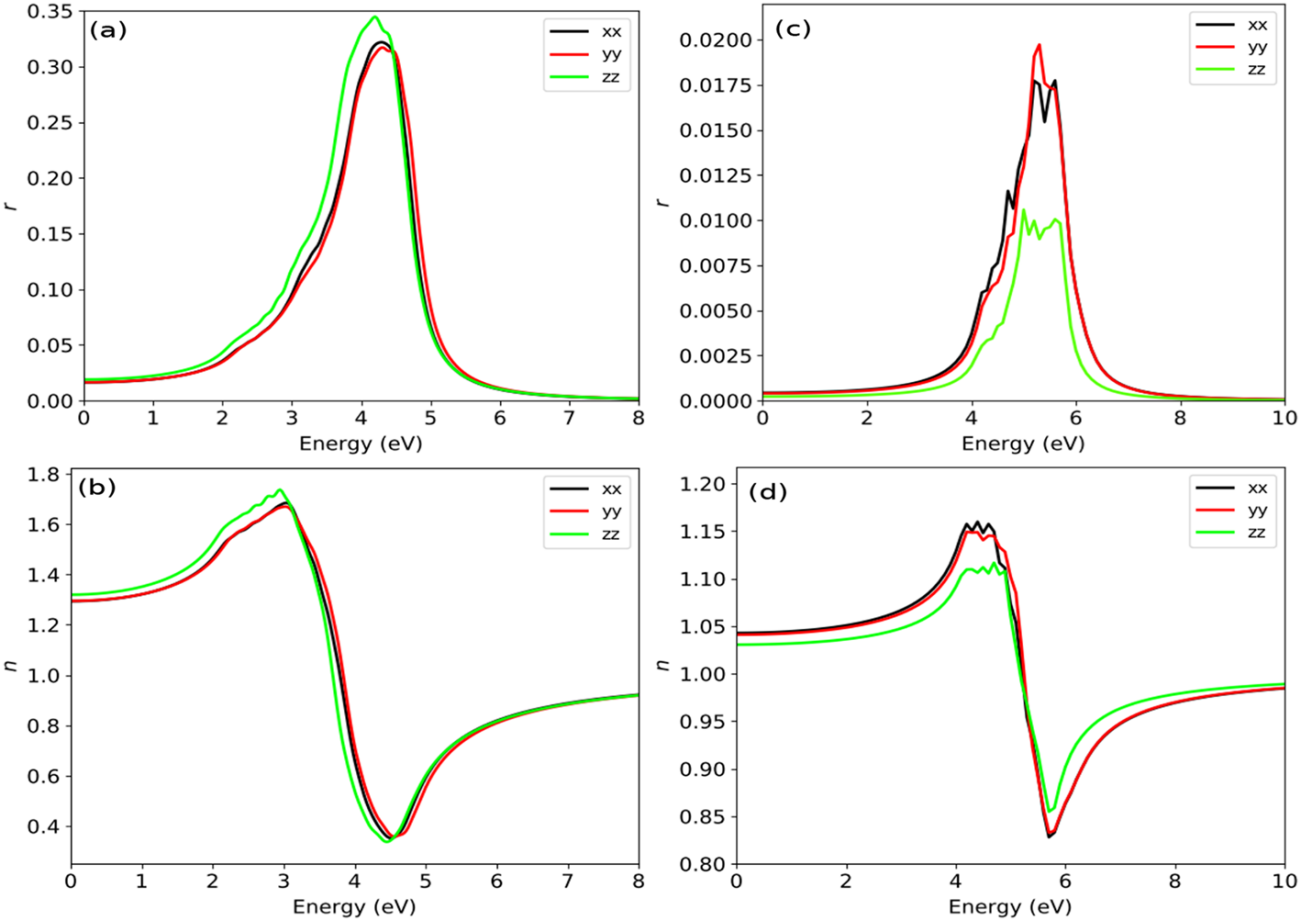
(**a**) Reflectivity (r) of bulk, (**b**) Refractive index (*n*) of bulk, (**c)**Reflectivity (r) of 001-surface, (**d**) Refractive index (*n*) of 001-surface.

The optical parameters of both bulk and surface like real part of dielectric function ($\varepsilon _1(\omega )$), imaginary part of dielectric function($\varepsilon _2(\omega )$), absorption coefficient($\alpha (\omega )$, reflectivity($r(\omega )$) and refractive index ($n(\omega )$) are presented in Figs. 7, 8 and 9. In all figures we have used black, red and green color lines to represent the polarization along *x*, *y* and *z*-axes, respectively. Fig.7a and b represent the real and imaginary part of dielectric function for the bulk system. The $\varepsilon _1(\omega )$ rises slowly and reach maximum at $\sim$2.35 eV, in which the dielectric polarization along *z*-axis predominate. After 2.5 eV the $\varepsilon _1(\omega )$ decreases sharply and drops below 0 at $\sim$3.0 eV. The drop of $\varepsilon _1(\omega )$ below zero gives negative value in which the incident photon beam is attenuated due to the dissipation of energy into the medium and giving rise to metallic behaviour. The drop of $\varepsilon _1(\omega )$ from maximum value can be relate to the rise of $\varepsilon _2(\omega )$, suggesting inter-band transition. The intensity of $\varepsilon _1(\omega )$ increases slowly after 3.0 eV and gives a constant intensity of $\sim$1.0 for all values of photon energy above 5.0 eV. The static value of real dielectric constant [$\varepsilon _1(0)$] is inversely related to the energy bandgap $E_g$ by;

10 $$\begin{aligned} \varepsilon _1(0)=1+\Big (\frac{\hbar \omega }{E_g} \Big )^2 \end{aligned}$$

From the above Eq. (10), the higher value of bandgap leads to the low value of $\varepsilon _1(0)$. It is well verified that the higher value of bandgap (3.82 eV) in the surface system gives far lower value of $\varepsilon _1(0)$=1.10, as compared to the bulk system [$\varepsilon _1(0)$=1.72]. The calculated values of $\varepsilon _1(0)$ along the different polarization direction is tabulated in Table 3. In $\varepsilon _2(\omega )$ spectra the maximum peak occurs at around 3.45 eV (see Fig. 7b). We have presented the $\varepsilon _2(\omega )$ spectra of 001 surface of CsPbBr$_3$ obtained from DFT-1/2 in Fig.7d. Unfortunately, we did not find any experimental result of $\varepsilon _2(\omega )$ spectra of the thin film of CsPbBr$_3$ for direct comparision. Therefore, the calculated $\varepsilon _2(\omega )$ is compared with the available result of nanocrystalline orthorhombic CsPbBr$_3$ calculated at different temperatures from high-resolution spectroscopic ellipsometry, high-resolution transmission electron microscopy and terahertz spectroscopy measurements^76^. The theoretical $\varepsilon _2(\omega )$ calculated from DFT-1/2 and experimental^76^ $\varepsilon _2(\omega )$ are plotted together in Fig. 7d for better comparision. Here, $\varepsilon _1(\omega )$ along *x* and *y*-axes predominates, due to the 2D nature of CsPbBr$_3$. The maximum value of $\varepsilon _1(\omega )$ occurs at $\sim$4.20 eV. It seems that the maximum peak of $\varepsilon _1(\omega )$ spectra has shifted towards the higher energy as compared to its bulk counter part. The highest peak of $\varepsilon _2(\omega )$ is located at $\sim$4.60 eV.

The absorption coefficient ($\alpha$) for both the bulk and the surface with respect to the photon energy and the wavelength is displayed in Fig. 8a–d. The positive tangent drawn on the $\alpha (\omega )$ cut the *x*-axis somewhere at $\sim$2.30 eV, which may be considered as an optical band gap (Fig. 8a). From, Fig. 8b, the estimated absorption onset is $\sim$540 nm, which is in good agreement with 554 nm^58^. Majhi et al., prepared the orthorhombic CsPbBr$_3$ from wet chemical synthesis method and studied the optoelectronic properties by UV–Vis and photoluminescence (PL) spectroscopy in which they reported the band-to-band transition at 554 nm (2.23 eV)^58^, which may be considered as an optical band gap. This spectra can be related to the first electron transition from top of the valence band to the bottom of conduction band along $\Gamma -\Gamma$ direction. For the bulk system an absorption peak of magnitude $\sim$4.5$\times$10$^5$ cm$^{-1}$ appears at $\sim$4.0 eV, has zero absorption intensity in the range of 0–2 eV and beyond 6.0 eV (see Fig. 8a). This proves that the for the bulk CsPbBr$_3$ the energy active window lies between 2.3 and 5.0 eV, having width of $\sim$2.70 eV. We have observed a reduce absorption peak in the case of surface system. The intensity of the peak for the surface is $\sim$1.58$\times$10$^5$ cm$^{-1}$ at $\sim$5.60 eV (see Fig.8c). The magnitude of $\alpha$= $\sim$1.58$\times$10$^5$ cm$^{-1}$ comprehended well with the experimental result^77^. The energy active window found in between 4 and 6.5 eV, giving rise to the window width of $\sim$2.5 eV. The window width for the bulk system is little high as compared to the surface by $\Delta W$=0.20 eV. The absorption onset is found at $\sim$340 nm (see Fig.8d). There is a shift in the absorption peak by $\sim$1.60 eV towards the higher energy while scaling from the infinite layers (bulk) to a single layer (surface). We have observed a prominent feature of a blue-shift in diminishing a structure size from bulk to surface(ultra thin film). It has already been shown that the perovskite with the slab thickness below 3.0 nm exhibit a blue shift [see Fig. 3 of Ref.^70^]. Most of the MAPbBr$_3$ perovskite nanoparticles of larger sizes (more than 50–100 nm) exhibit the influence of enhanced Mie modes preserving the blue shift despite the of feeble quantum confinement effect^75^. Electron energy-loss spectroscopy (EELS) reported the onset optical gap of around 2.4–2.5 eV for a 10 nm thick thin film of CsPbBr$_3$^59^. The thin film of CsPbBr$_3$ perovskite prepared from Single Source Thermal Ablation shows absorption onset at 530 nm^78^. Also, the CsPbBr$_3$ film prepared by 2-step sequential deposition, exhibit prominent absorbance peaks at 510–525 nm, with absorbance onset at 532 nm^79^.

**Table 3:** Static real part of dielectric function $\varepsilon _1(0)$and static refractive index *n*(0) of orthorhombic bulk and surface of CsPbBr$_3$.

Structure	$\varepsilon _1(0)$			*n*(0)		
	$\varepsilon _1^x(0)$	$\varepsilon _1^y(0)$	$\varepsilon _1^z(0)$	$n^x(0)$	$n^y(0)$	$n^z(0)$
Bulk	1.720	1.720	1.800	1.300	1.300	1.320
Surface	1.100	1.100	1.060	1.045	1.045	1.040

We have presented the calculated spectra of reflectivity and the refractive index for both the bulk and surface systems in Fig. 9a–d. At the initial part of the photon energy, the reflectivity spectra is very low and increases with the increase in the energy and ultimately give maximum reflectivity of $\sim$35.0% at $\sim$4.0 eV (see Fig. 9a). Meanwhile, the maximum reflectivity of the surface is only $\sim$2.0%, this may be due to its transparent behaviour within UV–Vis range of the electro-magnetic radiation Fig. 9c. The maximum surface reflectivity occurs at $\sim$5.60 eV. Fig. 9b and d, shows the refractive index of the bulk and the surface. The static refractive indices for the bulk system along *x* and *z*-direction are 1.72 and 1.80, respectively. This result is in good agreement with the previous result of 1.96^45,46^. From the static value, the $n(\omega )$ increases to reach the maximum value at $\sim$3.20 eV. Beyond 3.20 eV the spectra of $n(\omega )$ decreases rapidly and goes below 1.0 at 3.40 eV in the UV-region. If we refer to Fig. 7a, we can see the value of $\varepsilon _1(\omega )$ becomes negative at this value of energy ($\sim$3.40 eV). The presence of $n(\omega )$ value below 1.0 is nonphysical in which the phase velocity move faster than the group velocity (speed of light). This can be related to the occurrence of the plasmonic vibration with the plasmonic frequency ($\omega _p$) close to resonance frequency. The static refractive index of the surface is found to be *n*(0)=1.045 which is close to 1.0, indicating transparent behaviour within visible range of light. In case of the surface the plasmonic vibration lies at $\sim$5.2 eV.

## Conclusions

For the first time, we have carried out the electronic and optical properties of the orthorhombic bulk and 001-surface of CsPbBr$_3$ from DFT-1/2 approach. This work also performed to test the efficiency of DFT-1/2 in deriving the electronic and optical properties of inorganic perovskite. Herein, we report the efficiency of DFT-1/2 is as effective as that of higher order DFT like HSE hybrid functional in calculating the electronic bandgap. The calculated band gap is 2.36 eV in good agreement with the experiment. While, the ultra-thin surface slab of CsPbBr$_3$ open up the electronic bandgap by $\sim$42%. We report the decrease in bandgap while increasing the slab thickness of CsPbBr$_3$ film. The thin film of CsPbBr$_3$ exhibit tunability of the bandgap via the surface thickness modification. The presence of high value of absorption coefficient $\sim$4.5$\times$10$^5$ cm$^{-1}$ and $\sim$1.58$\times$10$^5$ cm$^{-1}$ in UV–Vis energy range for both the bulk and the surface, respectively. The tunability of energy bandgap offers remarkable optoelectronic properties in UV–Vis range making this material promising for optoelectronic applications.

## Data Availability

The data for this paper are available from D.P.R (dibya@pucollege.edu.in) & M.E (Meabas@ju.edu.sa).
